# Light-responsive rotaxane-based materials: inducing motion in the solid state

**DOI:** 10.3762/bjoc.19.64

**Published:** 2023-06-14

**Authors:** Adrian Saura-Sanmartin

**Affiliations:** 1 Departamento de Química Orgánica, Facultad de Química, Universidad de Murcia, 30100 Murcia, Spain

**Keywords:** light irradiation, light-responsive materials, mechanical bond, mechanically interlocked materials, rotaxanes

## Abstract

Light-responsive rotaxane-based solid-state materials are ideal scaffolds in order to develop smart materials due to the properties provided by the mechanical bond, such as control over the dynamics of the components upon application of external stimuli. This perspective aims to highlight the relevance of these materials, by pointing out recent examples of photoresponsive materials prepared from a rotaxanated architecture in which motion of the counterparts and/or macroscopic motion of the interlocked materials are achieved. Although further development is needed, these materials are envisioned as privileged scaffolds which will be used for different advanced applications in the area of molecular machinery.

## Introduction

Light turns out to be a suitable and tailorable stimulus in order to develop materials showing improved functionalities, such as those of smart materials [1–5]. The characteristics of light which lead to an increased interest of several researchers are: (i) the remote and simple switching tunability; (ii) the different possibilities by varying different parameters, including irradiation time, wavelength and intensity; and (iii) the clean and nonthreatening performance.

In search of new organic molecular materials, the development of a wide variety of suitable compounds is essential. The use of stimuli-responsive molecules has paved the way for the preparation of advanced functional materials [6–10]. In this scenario, mechanically interlocked molecules (MIMs) have postulated as ideal scaffolds [11]. In particular, rotaxanes and pseudorotaxanes have led to a greater number of applications due to their inherent dynamics and the switching possibility through a rational design [12]. Thus, rotaxane-based materials have attracted the interest of the scientific community due to their enhanced properties and functionalities [13–17].

Although great strides have been made in the development of photoresponsive rotaxanes [18–29], progress on interlocked materials working via photoirradiation is less abundant in the literature. However, photoswitchable rotaxane-based materials have shown very interesting machine-like operation modes, highlighting the macroscopic transport of iodomethane drops along an inclined gold surface caused by light-triggered variations in the polarophobicity of interlocked fumaramide-based films [30]. Another application is the functionalization of mesoporous silica nanoparticles with light-responsive rotaxane-based molecular shuttles to control the uptake and release of target molecules [31–34].

This perspective is focused on recent examples of light-responsive rotaxane-based solid-state materials in which dynamics of the components and/or macroscopic motion of the material are accomplished, dividing the article into three main sections: (i) photoresponsive rotaxane discrete crystals; (ii) photoresponsive rotaxane polymers; and (iii) photoresponsive metal-organic rotaxane frameworks (MORFs). Besides to analyze selected recent examples, a critical opinion on the state of the art is provided, including some future directions of this research field and postulating light-responsive solid-state rotaxane materials as tailorable scaffolds which will be used in a wide range of advanced applications.

## Discussion

### Photoresponsive rotaxane discrete crystals

A complete understanding of the crystallization mechanisms accompanied by a rational design can lead to the obtention of crystalline molecular materials which allow the dynamics of the counterparts to take place [35–37]. Indeed, the motion of the cyclic counterparts of rotaxanes in crystalline molecular solids has been studied [38–39]. Rotaxane crystals bearing ferrocene motifs experienced elongation and contraction along the axes in a rapid and reversible manner by simply turning on and off a laser light irradiation, thus providing enough free space in order to allow an effective molecular motion in the crystal [40–43].

**Figure 1:**
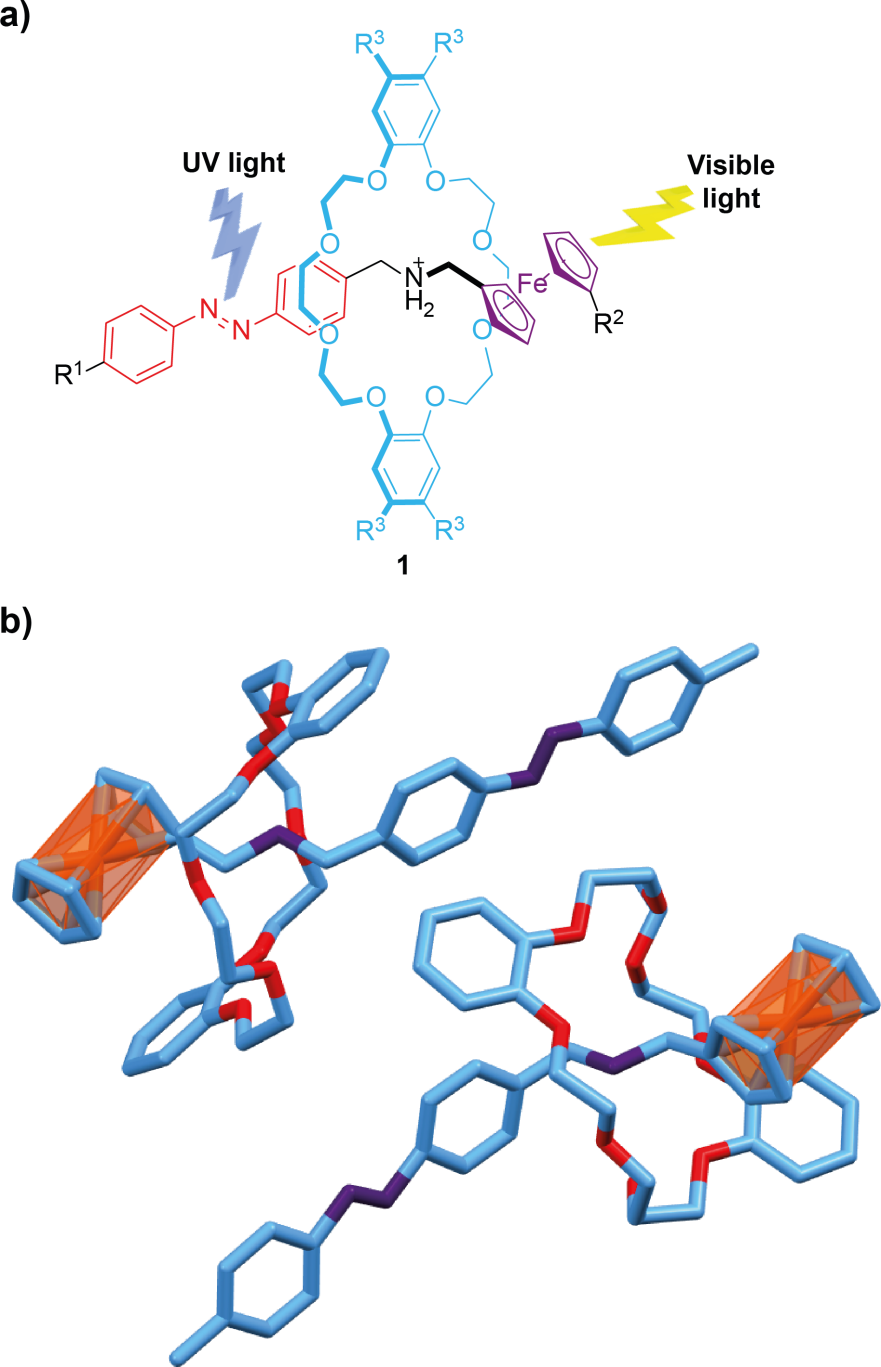
a) Chemical structure of pseudorotaxanes **1**; and (b) single-crystal X-ray structure of rotaxane **1a** (R^1^ = Me, R^2^ = R^3^ = H) showing two interlocked molecules of the crystalline array [44]. Colour key of the solid structure: light blue = carbon atoms; purple = nitrogen atoms; red = oxygen atoms; and orange = iron atoms. Hydrogen atoms are omitted for clarity.

The light-triggered crystal deformation of a series of [2]pseudo- and [3]pseudorotaxanes have been reported by Horie and coworkers [44]. It should be pointed out that although the examples highlighted in this section are pseudorotaxanes, the supramolecular interactions between the counterparts are retained, thus constituting stable intertwined species showing analogous properties to those of rotaxanes. The pseudorotaxanes **1** were constituted by a dibenzo-24-crown-8 cyclic component and an ammonium-based thread functionalized with azobenzene and ferrocene motifs (Figure 1a). The azobenzene scaffolds play a dual role, both as the engine transforming photoenergy into mechanical motion via *trans*/*cis* photoisomerization upon UV light input and as a modulator of the crystalline packing by varying the *para*-substituent R^1^, which leads to different flexibility. The crown ether derivative acts as a chassis in order to fix the thread. A ferrocenyl group attached at one of the ends of the linear component serves as a photosensitizer allowing the absorption of visible light. The different substitution induced different types of deformations, such as bending, jumping and curling, upon light irradiation. Interestingly, the bending of the crystals could proceed in different directions. As an example, crystals of pseudorotaxane **1a** (Figure 1b), having hydrogens as substituents R^2^ and R^3^ placed at the ferrocenyl motif and macrocycle, respectively, and a methyl group at the *para*-position of the ended-aromatic ring of the azobenzene motif (R^1^), experienced an upward bending using a 360 nm diode pump solid state laser irradiation source. By irradiating crystals of **1a** at 445 nm using a power of 4 mW, a reversible curling motion was observed. Flipping motions were induced enhancing the irradiation power to 12 mW. Noteworthy, these crystals showed a 9600 times higher weight ratio than its crystal weight due to the bending and expansion experienced by these crystalline molecular materials. These nanomachines are useful in the development of small-scale nanotechnological devices.

### Photoresponsive rotaxane polymers

Polymers represent an ideal support for the integration of rotaxane scaffolds [13,45]. Thus, advanced applications using polymers bearing rotaxanes have been reported [46–51]. The incorporation of light-responsive motifs in rotaxane-based polymers has also provided interesting properties which can be employed in some specific implementations.

**Figure 2:**
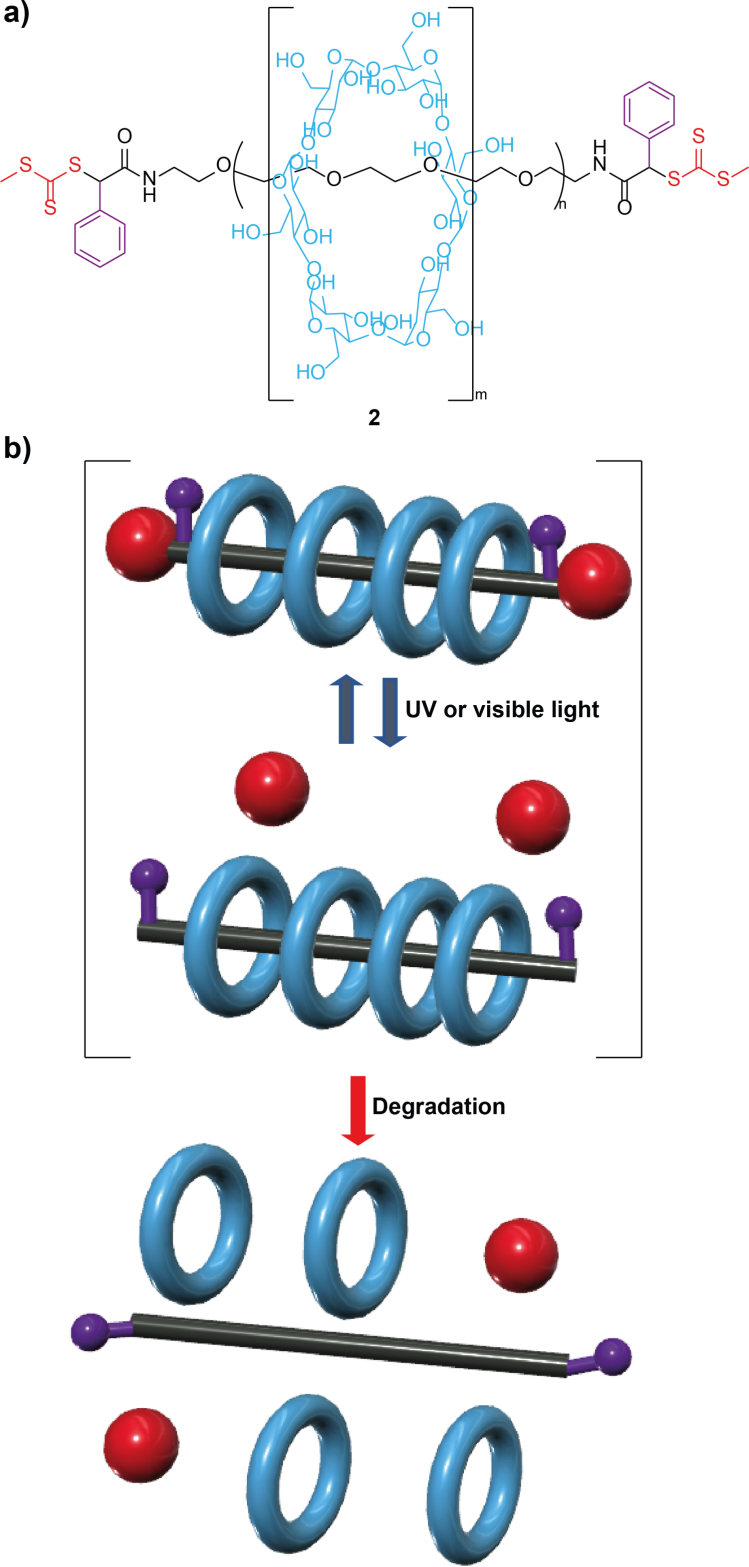
(a) Chemical structure of polyrotaxane **2**; and (b) cartoon representation of the light-triggered degradation of rotaxane polymer **2** [52]. The key colour of the cartoon representation is analogous to that of the chemical structures.

α-Cyclodextin-based polyrotaxanes **2** having trithiocarbonate stopper groups with an adjacent phenyl group were employed for the construction of visible light-degradable supramolecular gels (Figure 2a) [52]. Upon irradiation using a UV-light-emitting diode (LED) and a visible LED as sources, the reversible cleavage of the trithiocarbonate stoppers was accomplished, thus allowing the dethreading [53] of the wheels to take place by the shuttling of the macrocycles along the thread (Figure 2b). Interestingly, the viscosity of the gels was progressively reduced due to the decrease of the physical entanglement of the polymeric chains via photodegradation. Thus, the viscoelasticity of the rotaxane-based gels could be fine-tuned by modifying the irradiation time. This visible-light photodegradation of intertwined gels could lead to advanced functional materials avoiding the UV phototoxicity for biocompatible implementations, such as protein patterning and tissue engineering.

### Light-responsive metal-organic rotaxane frameworks

The integration of the mechanical bond into metal-organic frameworks (MOFs) [16,54] has allowed the dynamics of the different counterparts in the solid state, as well as some advanced applications [55–61].

**Figure 3:**
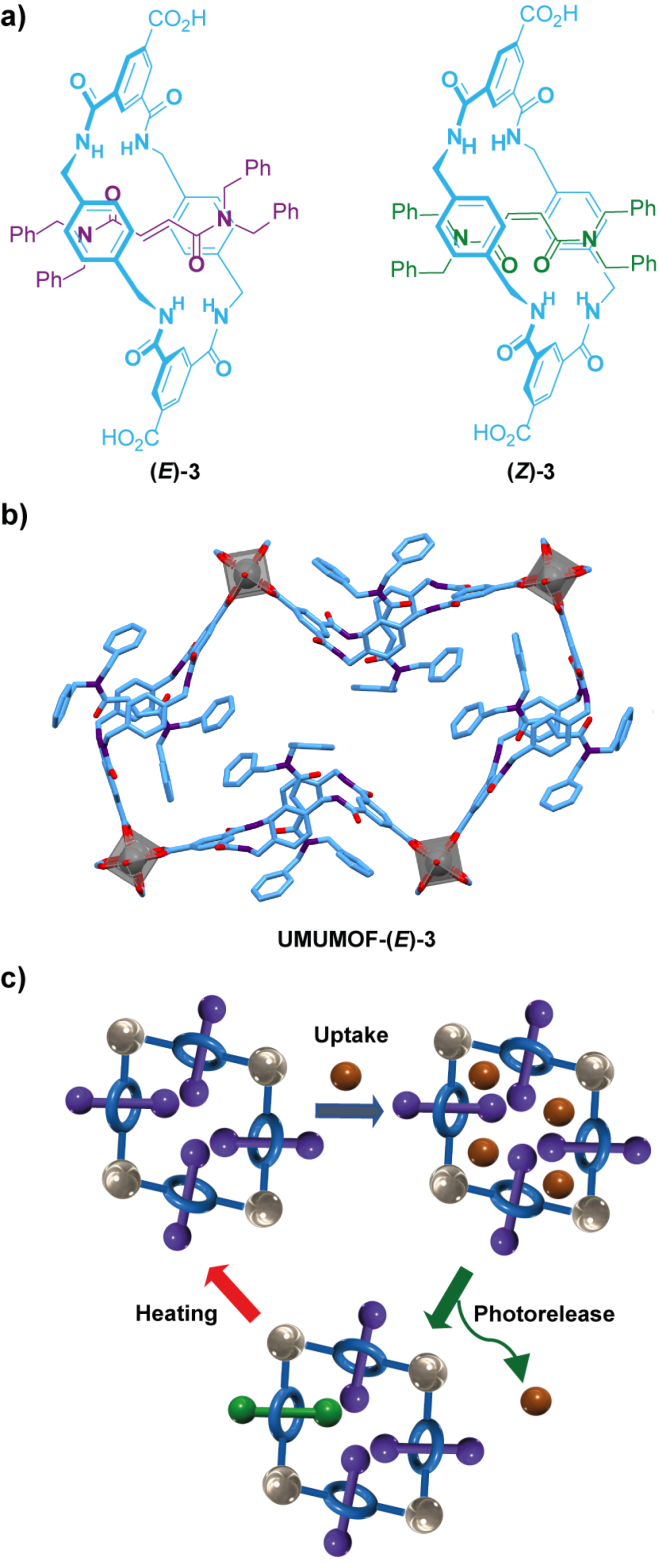
a) Chemical structures of rotaxanes **(*E*)-3** and **(*Z*)-3**; b) stick representation of the solid structure of **UMUMOF-(*E*)-3** showing a rhombohedral metallogrid; and (c) cartoon representation of the operation mode of **UMUMOF-(*E*)-3** as a molecular nanodispenser [62]. Colour key of the solid structure: light blue = carbon atoms; purple = nitrogen atoms; red = oxygen atoms; and grey = copper atoms. The key colour of the cartoon representation is analogous to that of the chemical structures.

Berna and colleagues prepared a copper-organic framework (**UMUMOF-(*E*)-3**) containing the interlocked fumaramide **(*E*)-3** as the organic ligand (Figure 3a) [62], forming rhombohedral grids connecting four different rotaxane derivatives to distinct copper-paddlewheel clusters (Figure 3b). Upon irradiation at 312 nm using a photoreactor equipped with UV lamps, 20% of the fumaramide stations were photoconverted into the corresponding intertwined maleamides **(*Z*)-3** in the solid state (Figure 3a), leading to an enhancement in the porosity of the metal-organic crystalline material. Noteworthy, a MOF having **(*Z*)-3** as the only ligand was also prepared, showing a faster rotational dynamics of the threads within the crystalline array compared to that of **UMUMOF-(*E*)-3** due to the decrease of the number of hydrogen bonds interactions between the counterparts, as determined by solid-state ^2^H NMR. Interestingly, **UMUMOF-(*E*)-3** was employed as a molecular nanodispenser of *para*-benzoquinone working through a cyclic operation mode which involved three steps (Figure 3c): (i) an uptake of the molecular cargo was firstly accomplished by immersing the metal-organic crystals in a 1.2 M solution of *para*-benzoquinone in chloroform which leads to the loading of a 9.82% w/w of quinone; (ii) photoirradiation at 312 nm over a period of 8 hours which leads to the complete release of the cargo by the partial photoconversion of the fumaramide motifs that change porosity and hydrogen bonding interactions of the counterparts; and (iii) recovery of the starting material through a thermal treatment, allowing the reusability of the nanodispenser. The molecular cargo release was also possible by immersing the loaded MOFs into different solvents, showing a clear dependence of the polarity of the solvent and the rate of the delivery process. This approach to incorporate photoresponsive rotaxanes within MOFs paves the way for the development of novel molecular machines operating in the solid state as response to light inputs.

**Figure 4:**
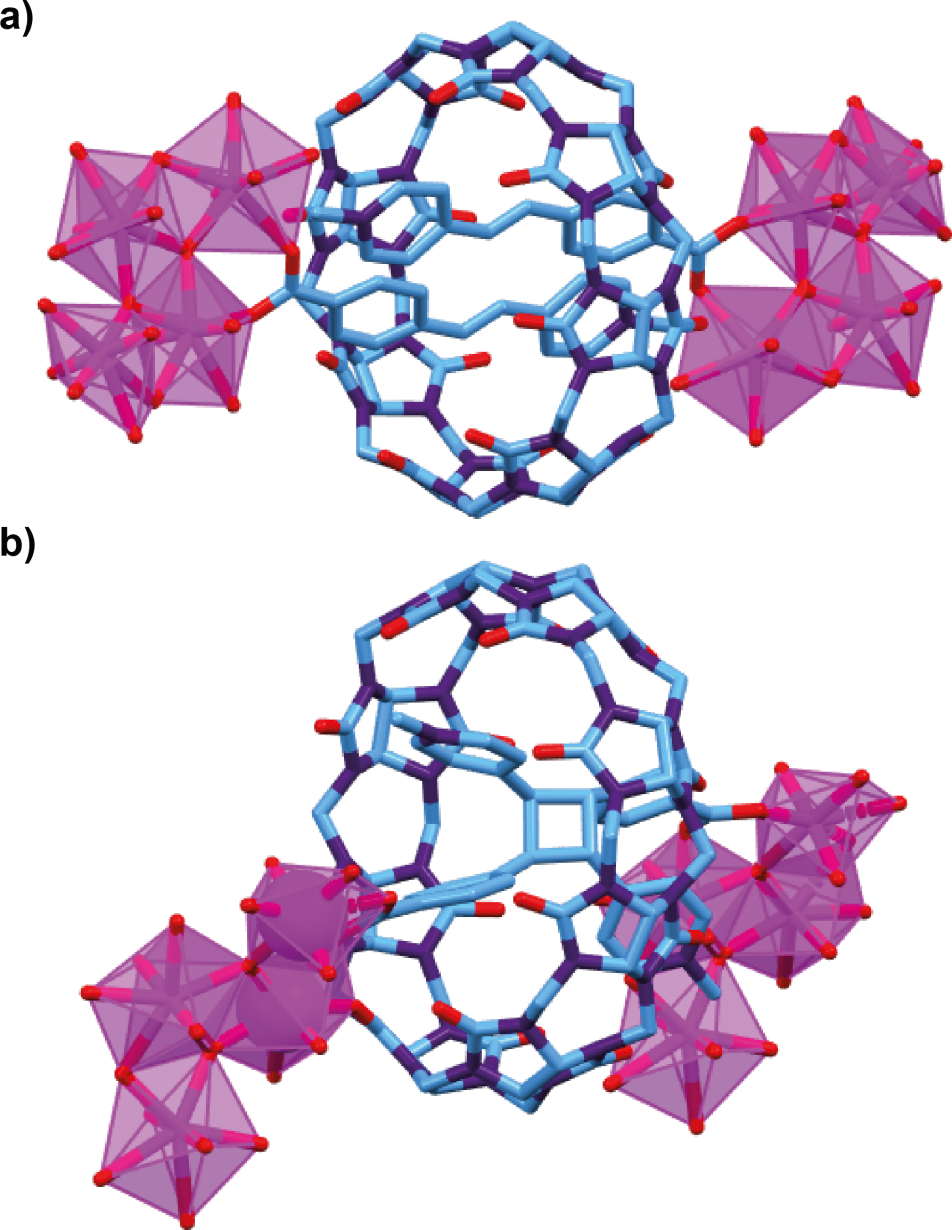
Stick representations of the solid structures of: (a) **U-CB[8]-MPyVB** showing an interlocked ligand connected to two uranium clusters; and (b) the intertwined photodimerized product within the crystalline array [63]. Colour key of the solid structure: light blue = carbon atoms; purple = nitrogen atoms; red = oxygen atoms; and magenta = uranium atoms.

In the examples discussed above, the light-activated motion of the counterparts controls the changes in the material scaffold, but macroscopic deformations of the material are also possible by photo-triggered reactions of different components within the interlocked arrangement. As an illustrative example, cucurbit[8]uril-based pseudorotaxanes having a pair of styrylpyridinium threads bearing carboxylic acid groups were employed in the preparation of the uranium-organic framework **U-CB[8]-MPyVB** (Figure 4a) [63]. The solid structure of the MOFs shows the styrene-based derivatives coordinated in an antiparallel manner through the carboxylic acid group placed at the end of each thread, thus avoiding the dethreading process. Two identical intertwined scaffolds were formed differing in the photoactivities due to different conformations. In the photoactive arrangement, a single-crystal-to-single-crystal regioselective [2 + 2] photodimerization reaction was accomplished by irradiating at 365 nm using a 6 W UV lamp. This light-triggered reaction led to the photoconversion of the threaded styrylpyridinium motifs into the corresponding interlocked cyclobutanes (Figure 4b). Interestingly, the changes in the crystalline array as a consequence of this photodimerization within the cucurbituril macrocycles also induced a macroscopic deformation of the metal-organic material, promoting a photomechanical bending of the crystals. This photomechanical deformation paves the way to the development of photoactuator devices leading to envision advanced applications in optomechanics and microrobotics.

### Outlook

The employment of light-responsive rotaxanes and pseudorotaxanes in the preparation of functional advanced materials leads to envision a promising future with the development of a plethora of improved functionalities and implementations. Although this research area is not well-explored yet, improved properties, as well as interesting applications have been reported. Thus, macroscopic changes in the solid-state materials have been carried out by light-induced responses of the rotaxane scaffolds, resulting in mechanical bending and other macroscopic transformations. But also, these changes have been exploited to perform some advanced applications, such as the development of molecular nanodispensers.

Despite the divergence of materials discussed in this perspective, there is a common link between these examples, the incorporation of rotaxane struts having photoresponsive units which trigger a motion in the solid state. The rotaxane discrete crystals experience a series of macroscopic deformations which are induced by the different dynamics of the counterparts upon light irradiation. In the highlighted polymeric example, a light input photodegrades the stoppers, allowing the dethreading process through the shuttling of the macrocycle along the linear component. The highlighted examples of MOFs include two different types of motion: (i) a different rotation rate of the threads by a light-driven exchange between geometric isomers of a rotaxane scaffold; and (ii) a macroscopic bending of the crystalline array by a photo-triggered dimerization reaction of two identical linear components within the macrocyclic counterpart. Thus, in all the discussed examples, a light input leads to the observation of motion into condensed phases, both at the level of intertwined components and in terms of macroscopic changes.

Despite these promising results reported so far, there are some issues which should be overcome, such as the photostationary equilibrium of some light-responsive intertwined materials and the industrial scale-up production usually hindered by the low yield of the interlocked molecules. Thus, future research efforts should be focused on the development of novel synthetic methodologies to access to such interlocked architecture, as well as the use of different templates [64–69]. The use of pyridyl-acyl hydrazone rotaxanes in the construction of light-responsive interlocked materials is envisioned as a promising approach to circumvent both issues, since these interlocked molecules are obtained in high yields (over 80%) and show great photoconversion (up to 98%) [18].

A rational design of the different components integrating the light-responsive interlocked materials turns out to be mandatory in order to develop fine-tuned machine-like operations in the solid state. One of the main directions of this research field should be focused on the integration of new photoswitchable scaffolds which allow both the formation of the intertwined species and the arrangement of the solid materials. In this regard, the functionalization of different templates already tested in solution chemistry is envisioned as the main strategy, incorporating different units that allow the integration within the corresponding materials. Thus, the incorporation of coordinating groups (i.e., pyridines or carboxylates) in the molecular design will allow the integration of such rotaxanes in MOFs [70], while other substituents will be necessary to prepare different materials [11].

Towards biocompatible applications [71], the use of visible light irradiation as input which leads to the desired function is a necessary requirement. In this scenario, the incorporation of photosensitizer motifs is a suitable strategy to allow such a performance. Towards this direction, one potential strategy is the approach followed by Feringa and co-workers [72], in which palladium-porphyrin photosensitizer-based struts were employed within a metal-organic material, allowing the use of green light as irradiation source because of the effective energy transfer between these struts and the photoresponsive linkers.

The use of ditopic interlocked building blocks, such as that employed to form mainly a cyclic hetero[4]pseudorotaxane from a self-complementary [2]rotaxane [73], is also envisioned as a strategy that will be employed in the future to dynamically change the properties of the material, leading to photochemically breakable and regenerative polymers showing a similar behaviour to that observed in diarylethene MOFs [74].

The range of materials in which light-responsive rotaxanes have been incorporated in order to induce motions of the counterparts in the solid state or macroscopic deformations of such materials is limited. Thus, the development of other materials incorporating photoresponsive rotaxanes is expected. In this scenario, this research field will take inspiration from the reported works focused on other types of materials incorporating rotaxanes and pseudorotaxanes, such as covalent organic frameworks [75], carbon nanotubes [17], metal-coordinated monolayers and multilayers [76–77], and dendrimers [78], among others.

The future development of light-responsive rotaxane-based materials will benefit from advances in the areas of photoresponsive materials and molecular machinery operating through light stimuli. Thus, the construction of smart materials by the rotaxane approach leads to envision a promising future in the area of photoresponsive materials incorporating molecular machines.

## References

[1] Katz J S, Burdick J A (2010). Macromol Biosci.

[2] Xiao K, Kong X-Y, Zhang Z, Xie G, Wen L, Jiang L (2016). J Photochem Photobiol, C.

[3] Marturano V, Cerruti P, Giamberini M, Tylkowski B, Ambrogi V (2017). Polymer.

[4] Saura-Sanmartin A (2022). Int J Mol Sci.

[5] Xu F, Feringa B L (2022). Adv Mater (Weinheim, Ger).

[6] Ramireddy R R, Raghupathi K R, Torres D A, Thayumanavan S (2012). New J Chem.

[7] Theato P, Sumerlin B S, O’Reilly R K, Epps T H (2013). Chem Soc Rev.

[8] Wei M, Gao Y, Li X, Serpe M J (2017). Polym Chem.

[9] Moulin E, Faour L, Carmona-Vargas C C, Giuseppone N (2019). Adv Mater (Weinheim, Ger).

[10] Liu Z, Zhang L, Sun D (2020). Chem Commun.

[11] Mena-Hernando S, Pérez E M (2019). Chem Soc Rev.

[12] Xue M, Yang Y, Chi X, Yan X, Huang F (2015). Chem Rev.

[13] Takata T (2020). ACS Cent Sci.

[14] Seale J S W, Feng Y, Feng L, Astumian R D, Stoddart J F (2022). Chem Soc Rev.

[15] Chen L, Sheng X, Li G, Huang F (2022). Chem Soc Rev.

[16] Saura-Sanmartin A, Pastor A, Martinez-Cuezva A, Cutillas-Font G, Alajarin M, Berna J (2022). Chem Soc Rev.

[17] López-Moreno A, Villalva J, Pérez E M (2022). Chem Soc Rev.

[18] Leigh D A, Marcos V, Nalbantoglu T, Vitorica-Yrezabal I J, Yasar F T, Zhu X (2017). J Am Chem Soc.

[19] Tron A, Pianet I, Martinez-Cuezva A, Tucker J H R, Pisciottani L, Alajarin M, Berna J, McClenaghan N D (2017). Org Lett.

[20] Yang L-P, Jia F, Cui J-S, Lu S-B, Jiang W (2017). Org Lett.

[21] Martinez-Cuezva A, Saura-Sanmartin A, Nicolas-Garcia T, Navarro C, Orenes R-A, Alajarin M, Berna J (2017). Chem Sci.

[22] Zhan T-G, Yin H-H, Zheng S-T, Lin W-C, Shen N-L, Cui J, Kong L-C, Liu L-J, Zhang K-D (2018). Chem Commun.

[23] Saura-Sanmartin A, Martinez-Cuezva A, Pastor A, Bautista D, Berna J (2018). Org Biomol Chem.

[24] Yu J-J, Zhao L-Y, Shi Z-T, Zhang Q, London G, Liang W-J, Gao C, Li M-M, Cao X-M, Tian H (2019). J Org Chem.

[25] Martinez-Cuezva A, Morales F, Marley G R, Lopez-Lopez A, Martinez-Costa J C, Bautista D, Alajarin M, Berna J (2019). Eur J Org Chem.

[26] Ogoshi T, Kotera D, Fa S, Nishida S, Kakuta T, Yamagishi T, Brouwer A M (2020). Chem Commun.

[27] Yang J-X, Li Z, Gu X-H, Zhan T-G, Cui J, Zhang K-D (2021). Tetrahedron.

[28] Lopez-Sanchez J, Alajarin M, Pastor A, Berna J (2021). J Org Chem.

[29] Chu C-W, Stares D L, Schalley C A (2021). Chem Commun.

[30] Berna J, Leigh D A, Lubomska M, Mendoza S M, Pérez E M, Rudolf P, Teobaldi G, Zerbetto F (2005). Nat Mater.

[31] Yan H, The C, Sreejith S, Zhu L, Kwok A, Fang W, Ma X, Nguyen K T, Korzh V, Zhao Y (2012). Angew Chem, Int Ed.

[32] Tarn D, Ferris D P, Barnes J C, Ambrogio M W, Stoddart J F, Zink J I (2014). Nanoscale.

[33] Martinez-Cuezva A, Valero-Moya S, Alajarin M, Berna J (2015). Chem Commun.

[34] Wang D, Wu S (2016). Langmuir.

[35] Martins M A P, Rodrigues L V, Meyer A R, Frizzo C P, Hörner M, Zanatta N, Bonacorso H G, Berna J, Alajarin M (2017). Cryst Growth Des.

[36] Orlando T, Salbego P R S, Farias F F S, Weimer G H, Copetti J P P, Bonacorso H G, Zanatta N, Hoerner M, Berna J, Martins M A P (2019). Eur J Org Chem.

[37] Orlando T, Salbego P R S, Taschetto C L R, Bonacorso H G, Zanatta N, Hoerner M, Martins M A P (2019). Cryst Growth Des.

[38] Horie M, Sassa T, Hashizume D, Suzaki Y, Osakada K, Wada T (2007). Angew Chem, Int Ed.

[39] Baggi G, Wilson B H, Dhara A, O’Keefe C A, Schurko R W, Loeb S J (2021). Chem Commun.

[40] Chen K-J, Tsai Y-C, Suzaki Y, Osakada K, Miura A, Horie M (2016). Nat Commun.

[41] Chen K-J, Chen P-L, Horie M (2017). Sci Rep.

[42] Cheng S-C, Chen K-J, Suzaki Y, Tsuchido Y, Kuo T-S, Osakada K, Horie M (2018). J Am Chem Soc.

[43] Chen K-J, Tan A C, Wang C-H, Kuo T-S, Chen P-L, Horie M (2019). Cryst Growth Des.

[44] Cheng S-C, Wang C-H, Lin Y-C, Tsuchido Y, Suzaki Y, Sei Y, Kuo T-S, Horie M (2020). ACS Appl Mater Interfaces.

[45] Takata T, Aoki D (2018). Polym J.

[46] Sawada J, Aoki D, Sun Y, Nakajima K, Takata T (2020). ACS Appl Polym Mater.

[47] Cai K, Shi Y, Zhuang G-W, Zhang L, Qiu Y, Shen D, Chen H, Jiao Y, Wu H, Cheng C (2020). J Am Chem Soc.

[48] Arisaka Y, Yui N (2021). Mater Lett.

[49] Cao Z, Wu D, Li M, Yang F, Li Z, An W, Jiang S, Zheng X, Niu C, Qu D (2022). Chin Chem Lett.

[50] Asthana D, Thomas D, Lockyer S J, Brookfield A, Timco G A, Vitorica-Yrezabal I J, Whitehead G F S, McInnes E J L, Collison D, Leigh D A (2022). Commun Chem.

[51] Thomas D, Tetlow D J, Ren Y, Kassem S, Karaca U, Leigh D A (2022). Nat Nanotechnol.

[52] Kang T W, Tamura A, Arisaka Y, Yui N (2021). Polym Chem.

[53] Saura-Sanmartin A (2023). Eur J Org Chem.

[54] Wilson B H, Loeb S J (2020). Chem.

[55] Vukotic N, Harris K J, Zhu K, Schurko R W, Loeb S J (2012). Nat Chem.

[56] Vukotic N, O’Keefe C A, Zhu K, Harris K J, To C, Schurko R W, Loeb S J (2015). J Am Chem Soc.

[57] McGonigal P R, Deria P, Hod I, Moghadam P Z, Avestro A-J, Horwitz N E, Gibbs-Hall I C, Blackburn A K, Chen D, Botros Y Y (2015). Proc Natl Acad Sci U S A.

[58] Wilson B H, Vojvodin C S, Gholami G, Abdulla L M, O’Keefe C A, Schurko R W, Loeb S J (2021). Chem.

[59] Xia T, Yu Z-Y, Gong H-Y (2021). Molecules.

[60] Feng L, Qiu Y, Guo Q-H, Chen Z, Seale J S W, He K, Wu H, Feng Y, Farha O K, Astumian R D (2021). Science.

[61] Li X, Xie J, Du Z, Yu R, Jia J, Chen Z, Zhu K (2022). Chem Commun.

[62] Saura-Sanmartin A, Martinez-Cuezva A, Bautista D, Marzari M R B, Martins M A P, Alajarin M, Berna J (2020). J Am Chem Soc.

[63] Geng J, Mei L, Liang Y, Yuan L, Yu J, Hu K, Yuan L, Feng W, Chai Z, Shi W (2022). Nat Commun.

[64] Crowley J D, Goldup S M, Lee A-L, Leigh D A, McBurney R T (2009). Chem Soc Rev.

[65] Evans N H (2019). Eur J Org Chem.

[66] Shahraki B T, Maghsoudi S, Fatahi Y, Rabiee N, Bahadorikhalili S, Dinarvand R, Bagherzadeh M, Verpoort F (2020). Coord Chem Rev.

[67] Heard A W, Goldup S M (2020). ACS Cent Sci.

[68] Joy F, Nizam A, Nair Y, Pillai R S, Devasia J, Nagella P (2022). Eur Polym J.

[69] Yu J, Gaedke M, Schaufelberger F (2023). Eur J Org Chem.

[70] Vukotic V N, Loeb S J (2012). Chem Soc Rev.

[71] Riebe J, Niemeyer J (2021). Eur J Org Chem.

[72] Danowski W, Castiglioni F, Sardjan A S, Krause S, Pfeifer L, Roke D, Comotti A, Browne W R, Feringa B L (2020). J Am Chem Soc.

[73] Saura-Sanmartin A, Nicolas-Garcia T, Pastor A, Quiñonero D, Alajarin M, Martinez-Cuezva A, Berna J (2023). Chem Sci.

[74] Sato H, Matsui T, Chen Z, Pirillo J, Hijikata Y, Aida T (2020). J Am Chem Soc.

[75] Das G, Sharma S K, Prakasam T, Gándara F, Mathew R, Alkhatib N, Saleh N, Pasricha R, Olsen J-C, Baias M (2019). Commun Chem.

[76] Schwarz F B, Heinrich T, Lippitz A, Unger W E S, Schalley C A (2016). Chem Commun.

[77] Schwarz F B, Heinrich T, Kaufmann J O, Lippitz A, Puttreddy R, Rissanen K, Unger W E S, Schalley C A (2016). Chem – Eur J.

[78] Wang X-Q, Li W-J, Wang W, Yang H-B (2021). Acc Chem Res.

